# Heuristic pairwise alignment of de Bruijn graphs to facilitate simultaneous transcript discovery in related organisms from RNA-Seq data

**DOI:** 10.1186/1471-2164-16-S11-S5

**Published:** 2015-11-10

**Authors:** Shuhua Fu, Aaron M Tarone, Sing-Hoi Sze

**Affiliations:** 1 Department of Biochemistry & Biophysics, Texas A&M University, College Station, TX 77843, USA; 2 Department of Entomology, Texas A&M University, College Station, TX 77843, USA; 3 Department of Computer Science and Engineering, Texas A&M University, College Station, TX 77843, USA

**Keywords:** RNA-Seq, de Bruijn graph, pairwise alignment

## Abstract

**Background:**

The advance of high-throughput sequencing has made it possible to obtain new transcriptomes and study splicing mechanisms in non-model organisms. In these studies, there is often a need to investigate the transcriptomes of two related organisms at the same time in order to find the similarities and differences between them. The traditional approach to address this problem is to perform *de novo *transcriptome assemblies to obtain predicted transcripts for these organisms independently and then employ similarity comparison algorithms to study them.

**Results:**

Instead of obtaining predicted transcripts for these organisms separately from the intermediate de Bruijn graph structures employed by *de novo *transcriptome assembly algorithms, we develop an algorithm to allow direct comparisons between paths in two de Bruijn graphs by first enumerating short paths in both graphs, and iteratively extending paths in one graph that have high similarity to paths in the other graph to obtain longer corresponding paths between the two graphs. These paths represent predicted transcripts that are present in both organisms.

We show that our algorithm recovers significantly more shared transcripts than traditional approaches by applying it to simultaneously recover transcripts in mouse against rat and in mouse against human from publicly available RNA-Seq libraries. Our strategy utilizes sequence similarity information within the paths that is often more reliable than coverage information.

**Conclusions:**

Our approach generalizes the pairwise sequence alignment problem to allow the input to be non-linear structures, and provides a heuristic to reliably recover similar paths from the two structures. Our algorithm allows detailed investigation of the similarities and differences in alternative splicing between the two organisms at both the sequence and structure levels, even in the absence of reference transcriptomes or a closely related model organism.

## Background

The advance of next generation sequencing technologies has made it possible to perform detailed studies of splicing mechanisms among eukaryotic organisms. A popular strategy is to first sequence their transcriptomes, then map the reads to reference databases. In non-model organisms, such reference databases are often unavailable, and *de novo *transcriptome assembly algorithms are employed to obtain predicted transcripts [1-4]. This is often achieved by first constructing a de Bruijn graph structure that contains all branching possibilities [5,6], then obtaining predicted transcripts based on coverage information along the paths.

**Figure 1:**
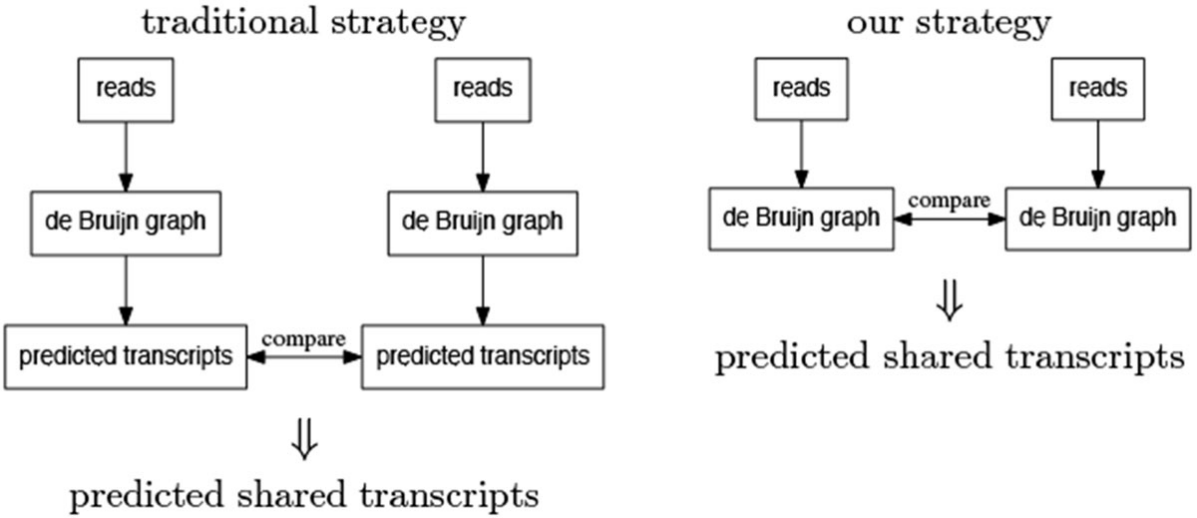
**Difference between traditional strategy and our strategy**.

In many of these studies, there is often a need to investigate the transcriptomes of two related organisms at the same time in order to study their similarities and differences. In these cases, RNA-Seq libraries are obtained from both organisms under different experimental conditions and the goal is to compare their transcriptome assemblies. The traditional approach to address this problem is to perform transcriptome assemblies to obtain predicted transcripts for the two organisms separately (see Figure 1). Similarity comparison algorithms such as BLAST [7] are then employed to extract corresponding transcripts that are shared in the two organisms.

Since predicted transcripts are constructed independently for each organism based on coverage information only, this strategy is often unreliable. To address this problem, we develop an algorithm to allow direct comparisons between paths in the two intermediate de Bruijn graph structures by an iterative extension strategy (see Figure 1). Since sequence similarity information is often more reliable, this strategy allows the direct extraction of shared transcripts based on evolutionary support.

## Methods

### De Bruijn graph

Given a set of reads and a parameter *k*, a de Bruijn graph is constructed by taking each *k*-mer that appears within the reads as a vertex. Two *k*-mers are connected by a directed edge if the (*k *− 1)-suffix of the first *k*-mer is the same as the (*k *− 1)-prefix of the second *k*-mer [5,6]. The de Bruijn graph implicitly assembles the reads by linking together the overlapping parts, and it is employed as the main intermediate structure by most short read assembly algorithms [8-12]. To obtain a more compact structure, each linear sequence of vertices that have no branches is collapsed into a single node that corresponds to contigs.

### Iterative extension

Given de Bruijn graphs *G*_1 _and *G*_2 _that correspond to transcriptome assemblies of two related organisms, we first apply BLAST to obtain similarity scores between each pair of nodes *u *from *G*_1 _and *v *from *G*_2_. We then start the iterative extension process as follows. For each node *u *from *G*_1_, we extract its most similar node *v *from *G*_2 _with *e*-value below a cutoff. If such a node *v *exists, we retain *u *as a single-node path. We extend *u *by one node along all its outgoing edges into multiple paths, and apply BLAST from each of these extended paths from *u *against *v*. If at least one of these extended paths gives a better *e*-value against *v*, we retain all the paths that have better *e*-values and continue to extend the top path that gives the best *e*-value. We repeat the procedure starting from this new path until the *e*-value no longer improves. Note that only one best direction is chosen since extending in more than one direction is very time-consuming. By starting from each node *u *in *G*_1 _independently, the probability of missing the real best path is reduced a lot.

**Figure 2:**
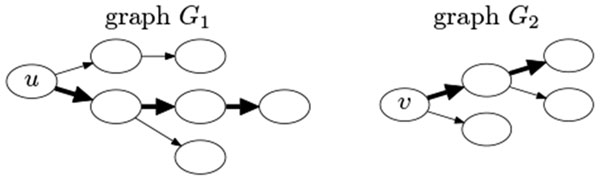
**Illustration of the iterative extension procedure**. The paths that are fully extended from *u *in *G*_1 _and from *v *in *G*_2 _are marked in bold, while the other retained paths with improved *e*-value are not marked.

After the above procedure, we have retained *u *and all the extended paths from *u *that have improved *e*-values, with the top path that gives the best *e*-value being fully extended. We then retain *v *as a single-node path and perform a similar extension process starting from *v *by extending it by one node along all its outgoing edges into multiple paths. We apply BLAST from each of these extended paths from *v *against all the retained paths from *u*. If at least one of these extended paths gives a better *e*-value, we retain all the paths that have better *e*-values and continue to extend the top path that gives the best *e*-value. Similar to above, we repeat the procedure starting from this new path until the *e*-value no longer improves to obtain a fully extended path and a set of retained paths from *v *that have improved *e*-values (see Figure 2).

We then repeat the entire extension procedure in turn in *G*_1 _and *G*_2 _by replacing *u* by the fully extended path from *u *and comparing against all the retained paths from *v*, and replacing *v* by the fully extended path from *v *and comparing against all the retained paths from *u*. The entire process is repeated until no more improvements can be made, and the algorithm is applied again by switching the role of *G*_1 _and *G*_2 _and repeating all the steps.

To obtain longer paths, we consider the retained paths from each node *u *and the retained paths from its twin node *u*′, in which *u*′ represents the reverse complementary sequence of *u *on the opposite strand. We merge the twin paths that are complementary to the retained paths from *u*′ with the retained paths from *u*, and keep those paths with improved *e*-values.

### Extraction of predicted transcripts

We consider all the retained paths in *G*_1 _as predicted transcripts in the first organism and all the retained paths in *G*_2 _as predicted transcripts in the second organism. Since the collection of all these retained paths can be very big, we only keep a path if it contains a node in the de Bruijn graph that is not covered by another path with a better *e*-value according to the top BLAST alignment. In this condition, a node is covered by a path if it contains the node itself or its twin node. To avoid a large number of incorrectly predicted isoforms, we remove paths with worse *e*-values so that each node in the de Bruijn graph along with its twin node appears at most 10 times within the final set of paths.

### Extraction of predicted shared transcripts

To obtain predicted shared transcripts that have correspondences between the two organisms, we apply BLAST from each predicted transcript in one organism against the set of all predicted transcripts in the other organism as database. We retain a predicted transcript as a predicted shared transcript if it appears both as a query with BLAST hits from one direction and as a subject BLAST hit in the other direction.

## Results and discussion

### Validation

We implement our algorithm Mutual as a postprocessing module of Velvet [10], which is a popular sequence assembly algorithm that returns a set of contigs along with the de Bruijn graph. We compare our performance to Oases [3], which uses output from Velvet to construct predicted transcripts. We validate our algorithm by applying it to simultaneously recover transcripts in mouse against rat and in mouse against human from publicly available RNA-Seq libraries at the sequence read archive [13], including two libraries from mouse in [14] (SRX017794), one library from rat in [15] (SRX076903), and four libraries from human in [16] (SRX011545). We perform quality trimming by removing all positions including and to the right of the first position that has a quality score of less than 15, resulting in a size of 1.3 G for the mouse libraries, 2.5 G for the rat libraries and 1.1 G for the human libraries.

We apply each algorithm over *k *= 25, 31, and over *k*-mer coverage cutoff *c *= 3, 5, 10. In our algorithm Mutual, iterative extension is applied twice with an *e*-value cutoff of 0.1 using the bl2seq (BLAST 2 Sequences) variant of BLAST, once with translated BLAST and once with nucleotide BLAST. Velvet and Oases are applied independently in each organism. Since Oases applies the coverage cutoff itself to obtain a de Bruijn graph by modifying Velvet's original de Bruijn graph without coverage cutoff, Mutual is applied on the two de Bruijn graphs given by Oases to obtain predicted transcripts.

To obtain predicted shared transcripts for both Oases and Mutual, we apply both translated BLAST and nucleotide BLAST with an *e*-value cutoff of 10^−7 ^or 10^−20^ from each predicted transcript in one organism against the set of all predicted transcripts in the other organism as database. The predicted transcripts that appear both as a query with BLAST hits from one direction and as a subject BLAST hit in the other direction are retained as predicted shared transcripts. To evaluate the accuracy of the predicted shared transcripts, we apply nucleotide BLAST to compare them against known mouse, rat or human transcriptome databases using the same *e*-value cutoff as the one used to obtain the transcripts, which is 10^−7 ^or 10^−20^. To assess the extent of translocated transcripts, we apply GMAP [17] to map the predicted shared transcripts to known mouse, rat or human genomes.

### Predicted transcripts

Tables 1 and 2 show that Mutual constructed less predicted transcripts than Oases. Note that the predicted transcripts from Mutual are obtained by extending similar paths that appear in the two organisms through iterative BLAST, while the predicted transcripts from Oases are obtained independently in each organism. The similarity constraints in Mutual ensure that a predicted transcript in one organism has a similar counterpart in the other organism, albeit with a loose *e*-value cutoff. The later reciprocal BLAST is needed to enforce more stringent *e*-value cutoffs. On the other hand, the predicted transcripts from Oases have no such constraints, and reciprocal BLAST is used to obtain shared transcripts.

**Table 1:** Comparisons of the number of predicted transcripts in the test on mouse against rat from Oases and from Mutual over different values of *k *and *k*-mer coverage cutoff *c*.

	mouse		rat	
***k*_*c***	**Oases**	**Mutual**	**Oases**	**Mutual**

25_3	51218	40657	100317	56409
25_5	27873	18511	33396	22538
25_10	10557	6104	7669	5639

31_3	48841	29778	82090	38141
31_5	25947	14073	28047	15981
31_10	8224	3954	5145	3485

Note that these numbers are not directly comparable between Oases and Mutual since the predicted transcripts from Mutual are obtained by extending similar paths that appear in the two organisms with an *e*-value cutoff of 0.1 from bl2seq, while the predicted transcripts from Oases are obtained independently in each organism without such constraints.

**Table 2:** Comparisons of the number of predicted transcripts in the test on mouse against human.

	mouse		human	
***k*_*c***	**Oases**	**Mutual**	**Oases**	**Mutual**

25_3	51218	34514	49579	36268
25_5	27873	18561	25911	17519
25_10	10557	7020	7672	5405

31_3	48841	23510	35993	23263
31_5	25947	13433	20396	12867
31_10	8224	4358	4705	3182

Notations are the same as in Table 1.

### Predicted shared transcripts

When compared to Tables 1 and 2, Tables 3 and 4 show that only a small percentage of predicted transcripts were shared in the two organisms, with a smaller decrease by Mutual than by Oases. The decrease by Mutual is due to more stringent *e*-value cutoffs, while the decrease by Oases is due to imposing similarity constraints between the two organisms. While the actual amount of predicted shared transcripts that can be recovered depends on the size of libraries, the evolutionary distance between the two organisms and the experimental conditions, Tables 3 and 4 show that Mutual recovered more predicted shared transcripts than Oases. Almost all these predicted shared transcripts are found in the corresponding known transcriptome database, with comparable percentages between Mutual and Oases. The percentages are lower in rat, probably due to the fact that the rat genome is less well annotated. The number of predicted shared transcripts decreases as the assembly parameters become more stringent, but these transcripts are of higher quality.

**Table 3:** Comparisons of the number of predicted shared transcripts (shared) and the number of predicted shared transcripts that have BLAST hits from each organism to its known transcriptome database (found) in the test on mouse against rat from Oases and from Mutual over different values of *k *and *k*-mer coverage cutoff *c* and over different *e*-value cutoffs 10^−^^7 ^and 10^−2^^0 ^.

	mouse(10^−7 ^)					rat(10^−7 ^)			
	**Oases**		**Mutual**			**Oases**		**Mutual**	
***k*_*c***	**shared**	**found**	**shared**	**found**	***k*_*c***	**shared**	**found**	**shared**	**found**

25_3	27671	26756 (97%)	35230	34011 (97%)	25_3	24489	21844 (89%)	39287	34298 (87%)
25_5	12729	12366 (97%)	14924	14520 (97%)	25_5	10092	9245 (92%)	15287	13639 (89%)
25_10	3955	3823 (97%)	4589	4465 (97%)	25_10	2994	2835 (95%)	3955	3705 (94%)

31_3	22635	22046 (97%)	25035	24396 (97%)	31_3	20917	19008 (91%)	27484	24744 (90%)
31_5	10229	10028 (98%)	11039	10825 (98%)	31_5	8398	7815 (93%)	11225	10332 (92%)
31_10	2597	2545 (98%)	2871	2815 (98%)	31_10	2013	1939 (96%)	2489	2382 (96%)

	**mouse(10^−20 ^)**					**rat(10^−20 ^)**			

	**Oases**		**Mutual**			**Oases**		**Mutual**	
***k*_*c***	**shared**	**found**	**shared**	**found**	***k*_*c***	**shared**	**found**	**shared**	**found**

25_3	22936	22290 (97%)	28705	27881 (97%)	25_3	19282	17719 (92%)	29923	26898 (90%)
25_5	10904	10608 (97%)	12648	12336 (98%)	25_5	8242	7669 (93%)	12087	10999 (91%)
25_10	3377	3253 (96%)	3901	3790 (97%)	25_10	2510	2388 (95%)	3254	3070 (94%)

31_3	18052	17627 (98%)	20026	19567 (98%)	31_3	15835	14699 (93%)	20943	19264 (92%)
31_5	8429	8261 (98%)	9157	8964 (98%)	31_5	6623	6218 (94%)	8886	8251 (93%)
31_10	2196	2150 (98%)	2438	2386 (98%)	31_10	1681	1629 (97%)	2041	1959 (96%)

The number in parentheses is the percentage of predicted shared transcripts that have BLAST hits from each organism to its known transcriptome database.

**Table 4:** Comparisons of the number of predicted shared transcripts and the number of predicted shared transcripts that have BLAST hits from each organism to its known transcriptome database in the test on mouse against human.

	mouse(10^−7^)					human(10^−7 ^)			
	**Oases**		**Mutual**			**Oases**		**Mutual**	
***k*_*c***	**shared**	**found**	**shared**	**found**	***k*_*c***	**shared**	**found**	**shared**	**found**

25_3	20763	20406 (98%)	25630	25189 (98%)	25_3	22499	22084 (98%)	28364	27911 (98%)
25_5	11914	11685 (98%)	12956	12784 (99%)	25_5	12037	11786 (98%)	12806	12643 (99%)
25_10	4644	4520 (97%)	5226	5114 (98%)	25_10	3844	3762 (98%)	4121	4047 (98%)

31_3	14631	14440 (99%)	16226	16041 (99%)	31_3	16498	16348 (99%)	18482	18318 (99%)
31_5	8351	8241 (99%)	8920	8825 (99%)	31_5	9250	9171 (99%)	9841	9753 (99%)
31_10	2727	2686 (98%)	2924	2887 (99%)	31_10	2326	2308 (99%)	2438	2420 (99%)

	**mouse(10^−20 ^)**					**human(10^−20 ^)**			

	**Oases**		**Mutual**			**Oases**		**Mutual**	
***k*_*c***	**shared**	**found**	**shared**	**found**	***k*_*c***	**shared**	**found**	**shared**	**found**
25_3	15532	15335 (99%)	18418	18165 (99%)	25_3	17014	16799 (99%)	19840	19558 (99%)
25_5	9534	9356 (98%)	10249	10137 (99%)	25_5	9718	9541 (98%)	10120	10000 (99%)
25_10	3965	3854 (97%)	4452	4358 (98%)	25_10	3344	3278 (98%)	3593	3529 (98%)

31_3	10165	10045 (99%)	11250	11127 (99%)	31_3	12052	11960 (99%)	13138	13043 (99%)
31_5	6262	6183 (99%)	6728	6654 (99%)	31_5	7267	7216 (99%)	7615	7557 (99%)
31_10	2245	2209 (98%)	2419	2385 (99%)	31_10	2003	1989 (99%)	2083	2069 (99%)

Notations are the same as in Table 3.

### Top BLAST hits to databases

By applying BLAST from each set of predicted shared transcripts in each organism to its known transcriptome database, Tables 5 and 6 show that Mutual recovered more shared transcripts than Oases, with many more shared transcripts recovered when the assembly parameters are less stringent. When compared to Tables 3 and 4, there is an effect of diminishing returns since a few thousand more predicted shared transcripts are needed to recover a few hundred more shared transcripts.

**Table 5:** Comparisons of the number of top unique BLAST hits to different transcripts from each set of predicted shared transcripts in each organism to its known transcriptome database in the test on mouse against rat from Oases and from Mutual over different values of *k *and *k*-mer coverage cutoff *c* and over different *e*-value cutoffs 10^−^^7 ^and 10^−20 ^.

10^−7^	mouse		rat		10^−20^	mouse		rat	
***k_c***	**Oases**	**Mutual**	**Oases**	**Mutual**	***k_c***	**Oases**	**Mutual**	**Oases**	**Mutual**

25_3	7780	8349 (+569)	7382	8061 (+679)	25_3	7035	7547 (+512)	6608	7148 (+540)
25_5	5310	5563 (+253)	4863	5158 (+295)	25_5	4715	4929 (+214)	4319	4538 (+219)
25_10	2361	2463 (+102)	2011	2094 (+83)	25_10	2008	2094 (+86)	1769	1833 (+64)

31_3	6645	6854 (+209)	6392	6660 (+268)	31_3	5780	5997 (+217)	5527	5802 (+275)
31_5	4286	4368 (+82)	3933	4103 (+170)	31_5	3713	3804 (+91)	3454	3557 (+103)
31_10	1705	1740 (+35)	1462	1517 (+55)	31 10	1443	1484 (+41)	1287	1320 (+33)

Only the top hit with *e*-value below the cutoff is considered. The number in parentheses is the change by Mutual over Oases.

**Table 6:** Comparisons of the number of top unique BLAST hits to different transcripts from each set of predicted shared transcripts in each organism to its known transcriptome database in the test on mouse against human.

10^−7^	mouse		human		10^−20^	mouse		human	
***k*_*c***	**Oases**	**Mutual**	**Oases**	**Mutual**	***k*_*c***	**Oases**	**Mutual**	**Oases**	**Mutual**

25_3	7090	7474 (+384)	7123	7548 (+425)	25_3	6169	6402 (+233)	6317	6539 (+222)
25_5	5308	5392 (+84)	5244	5318 (+74)	25_5	4666	4700 (+34)	4679	4696 (+17)
25_10	2781	2818 (+37)	2591	2612 (+21)	25_10	2452	2476 (+24)	2376	2385 (+9)

31_3	5490	5647 (+157)	5198	5387 (+189)	31_3	4421	4557 (+136)	4416	4547 (+131)
31_5	3918	3971 (+53)	3662	3732 (+70)	31_5	3221	3275 (+54)	3180	3222 (+42)
31_10	1796	1805 (+9)	1573	1594 (+21)	31_10	1531	1540 (+9)	1403	1410 (+7)

Notations are the same as in Table 5.

### Length distribution of transcripts

**Figure 3:**
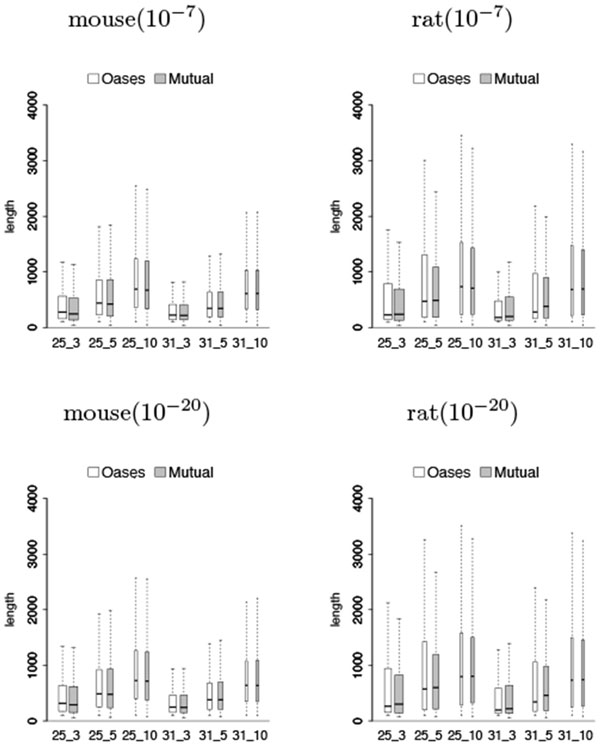
**Length distribution of predicted shared transcripts in the test on mouse against rat from Oases and from Mutual over different values of *k *and *k*-mer coverage cutoff *c* (represented by *k_c*) and over different *e*-value cutoffs 10^−7^ and 10^−20 ^**. The width of each box is proportional to the square root of the size of each group, while outliers are ignored.

**Figure 4:**
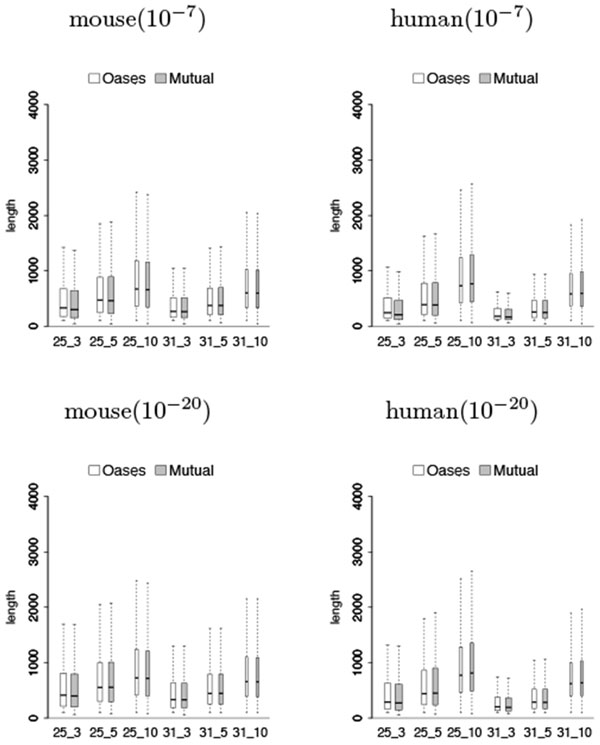
**Length distribution of predicted shared transcripts in the test on mouse against human**. Notations are the same as in Figure 3.

Figures 3 and 4 show that the lengths of predicted shared transcripts recovered by Mutual were comparable to the ones recovered by Oases, which are slightly shorter in mouse but have slightly higher medians in rat. These transcripts are generally longer when the *k*-mer coverage cutoff *c* increases.

### Recovery of full length transcripts

The situation is different when considering predicted shared transcripts that are close to full length. Tables 7 and 8 show that Mutual recovered more or a comparable number of 80% full length transcripts as Oases when the assembly parameters are more stringent, and less 80% full length transcripts than Oases when the assembly parameters are less stringent. Although Mutual performs worst in rat that recovers less 80% full length transcripts than Oases, its predicted shared transcripts have slightly higher median lengths when considering all the transcripts together (see Figure 3), instead of just the ones that are 80% full length transcripts.

**Table 7:** Comparisons of the number of predicted shared transcripts that are 80% full length transcripts in the test on mouse against rat from Oases and from Mutual over different values of *k *and *k*-mer coverage cutoff *c *and over different *e*-value cutoffs 10^−^^7 ^and 10^−2^^0 ^.

10^−7 ^	mouse		rat		10^−20 ^	mouse		rat	
***k*_*c***	**Oases **	**Mutual **	**Oases **	**Mutual **	***k*_*c***	**Oases **	**Mutual **	**Oases **	**Mutual **

25_3	1900	1840 (−60)	2066	1777 (−289)	25_3	1802	1743 (−59)	1870	1611 (−259)
25_5	1705	1677 (−28)	1739	1581 (−158)	25_5	1595	1561 (−34)	1577	1429 (−148)
25_10	1119	1097 (−22)	862	848 (−14)	25_10	984	975 (−9)	798	788 (−10)

31_3	1144	1158 (+14)	1407	1179 (−228)	31_3	1061	1077 (+16)	1226	1042 (−184)
31_5	1054	1062 (+8)	1240	1095 (−145)	31_5	966	990 (+24)	1092	978 (−114)
31_10	719	724 (+5)	662	662 (0)	31_10	638	646 (+8)	607	602 (−5)

These transcripts are the ones in which 80% of the coding region is included in the best BLAST alignment from each organism to its known transcriptome database. The number in parentheses is the change by Mutual over Oases.

**Table 8:** Comparisons of the number of predicted shared transcripts that are 80% full length transcripts in the test on mouse against human.

10^−7 ^	mouse		human		10^−20 ^	mouse		human	
***k*_*c***	**Oases **	**Mutual **	**Oases **	**Mutual **	***k*_*c***	**Oases **	**Mutual **	**Oases **	**Mutual **

25_3	1851	1808 (−43)	1529	1553 (+24)	25_3	1733	1686 (−47)	1450	1477 (+27)
25_5	1716	1666 (−50)	1534	1536 (+2)	25_5	1605	1552 (−53)	1454	1459 (+5)
25_10	1250	1241 (−9)	1178	1183 (+5)	25_10	1124	1112 (−12)	1114	1126 (+12)

31_3	1085	1099 (+14)	739	746 (+7)	31_3	995	1008 (+13)	686	700 (+14)
31_5	1009	1018 (+9)	734	736 (+2)	31_5	923	932 (+9)	678	683 (+5)
31_10	720	723 (+3)	627	628 (+1)	31_10	654	656 (+2)	579	585 (+6)

Notations are the same as in Table 7.

### Presence of translocated transcripts

As reported by GMAP, Tables 9 and 10 show that Mutual recovered a much larger number of predicted shared transcripts that are uniquely mapped than Oases, while at the same time returning more translocated transcripts that can be considered to be errors due to their rare occurrences [18]. The ratio of the number of translocated transcripts to the number of uniquely mapped transcripts is at most about twice as much for Mutual when compared to Oases. This ratio increases when *k *decreases or when the *k*-mer coverage cutoff *c *increases.

**Table 9:** Comparisons of the number of predicted shared transcripts that are uniquely mapped (unique) or translocated (transloc) as reported by GMAP in the test on mouse against rat from Oases and from Mutual over different values of *k *and *k*-mer coverage cutoff *c *and over different *e*-value cutoffs 10^−^^7 ^and 10^−2^^0 ^.

	mouse(10^−7^)					rat(10^−7^)			
	**Oases **		**Mutual **			**Oases **		**Mutual **	

***k*_*c***	**unique **	**transloc **	**unique **	**transloc **	***k*_*c***	**unique **	**transloc **	**unique **	**transloc **
25_3	24635	599 (0.024)	30713	1475 (0.048)	25_3	21335	986 (0.046)	33566	2237 (0.067)
25_5	10718	436 (0.041)	12071	1011 (0.084)	25_5	8509	438 (0.051)	12676	971 (0.077)
25_10	2913	218 (0.075)	3197	409 (0.128)	25_10	2353	122 (0.052)	3042	257 (0.084)

31_3	20360	242 (0.012)	22229	483 (0.022)	31_3	18236	497 (0.027)	23818	795 (0.033)
31_5	8778	189 (0.022)	9263	388 (0.042)	31_5	7132	251 (0.035)	9453	388 (0.041)
31_10	1914	99 (0.052)	2026	176 (0.087)	31_10	1553	65 (0.042)	1888	113 (0.060)

	**mouse(10^−20^)**						**rat(10^−20^)**		

	**Oases **		**Mutual **			**Oases**		**Mutual**	

***k*_*c***	**unique **	**transloc **	**unique **	**transloc **	***k*_*c***	**unique **	**transloc **	**unique **	**transloc **
25_3	20209	544 (0.027)	24662	25 3	25_3	16880	746 (0.044)	25851	1536 (0.059)
25_5	9070	396 (0.044)	10067	25 5	25_5	7021	332 (0.047)	10097	718 (0.071)
25_10	2431	188 (0.077)	2631	25 10	25_10	1977	98 (0.050)	2499	214 (0.086)

31_3	16077	213 (0.013)	17610	31 3	31_3	13866	376 (0.027)	18290	516 (0.028)
31_5	7136	156 (0.022)	7538	31 5	31_5	5656	177 (0.031)	7572	243 (0.032)
31_10	1590	85 (0.053)	1701	31 10	31_10	1299	51 (0.039)	1559	83 (0.053)

The number in parentheses is the ratio of the number of translocated transcripts to the number of uniquely mapped transcripts.

**Table 10:** Comparisons of the number of predicted shared transcripts that are uniquely mapped or translocated as reported by GMAP in the test on mouse against human.

	mouse(10^−7^)					human(10^−7^)			
	**Oases**		**Mutual**			**Oases**		**Mutual**	

***k*_*c***	**unique**	**transloc**	**unique**	**transloc**	***k*_*c***	**unique**	**transloc**	**unique**	**transloc**
25_3	18157	531 (0.029)	21931	1209 (0.055)	25_3	19912	224 (0.011)	25142	592 (0.024)
25_5	10036	393 (0.039)	10760	763 (0.071)	25_5	10353	150 (0.014)	11088	334 (0.030)
25_10	3582	203 (0.057)	3838	420 (0.109)	25_10	3114	78 (0.025)	3281	221 (0.067)

31_3	12899	196 (0.015)	14105	370 (0.026)	31_3	14748	65 (0.004)	16499	126 (0.008)
31_5	7084	147 (0.021)	7392	302 (0.041)	31_5	8101	43 (0.005)	8536	94 (0.011)
31_10	2029	93 (0.046)	2095	167 (0.080)	31_10	1858	30 (0.016)	1919	58 (0.030)

	**mouse(10^−20^)**					**human(10^−20^)**			

	**Oases**		**Mutual**			**Oases**		**Mutual**	

***k*_*c***	**unique**	**transloc**	**unique**	**transloc**	***k*_*c***	**unique**	**transloc**	**unique**	**transloc**
25_3	13313	499 (0.037)	15285	1073 (0.070)	25_3	14928	195 (0.013)	17259	518 (0.030)
25_5	7877	373 (0.047)	8286	713 (0.086)	25_5	8301	130 (0.016)	8638	315 (0.036)
25_10	2980	188 (0.063)	3152	400 (0.127)	25_10	2699	73 (0.027)	2822	211 (0.075)

31_3	8736	181 (0.021)	9504	330 (0.035)	31_3	10690	57 (0.005)	11621	106 (0.009)
31_5	5183	137 (0.026)	5408	281 (0.052)	31_5	6325	35 (0.006)	6580	84 (0.013)
31_10	1618	91 (0.056)	1671	161 (0.096)	31_10	1591	19 (0.012)	1623	55 (0.034)

Notations are the same as in Table 9.

### Accuracy of transcript reconstruction

**Figure 5:**
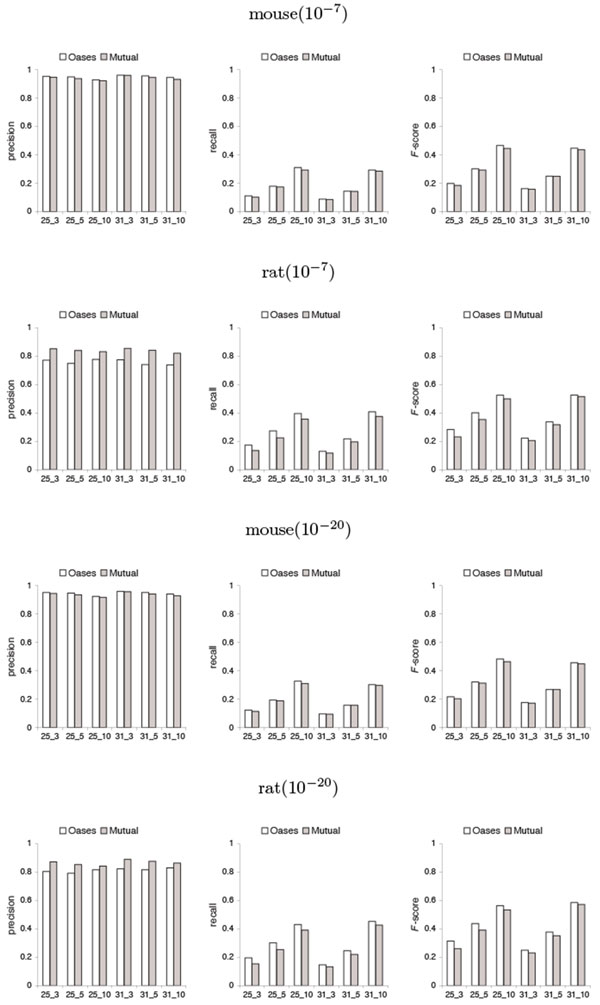
**Precision, recall and *F*-score with respect to the accuracy of shared transcript reconstruction in the test on mouse against rat from Oases and from Mutual over different values of *k *and *k*-mer coverage cutoff *c *(represented by *k*_*c*) and over different *e*-value cutoffs 10^−7 ^and 10^−20 ^**. Precision is defined to be the fraction of query positions from predicted shared transcripts that are included in BLAST alignments from each organism to its known transcriptome database. Recall is defined to be the fraction of subject positions from database sequences that are included in BLAST alignments from each organism to its known transcriptome database. *F*-score is the harmonic mean of precision and recall.

**Figure 6:**
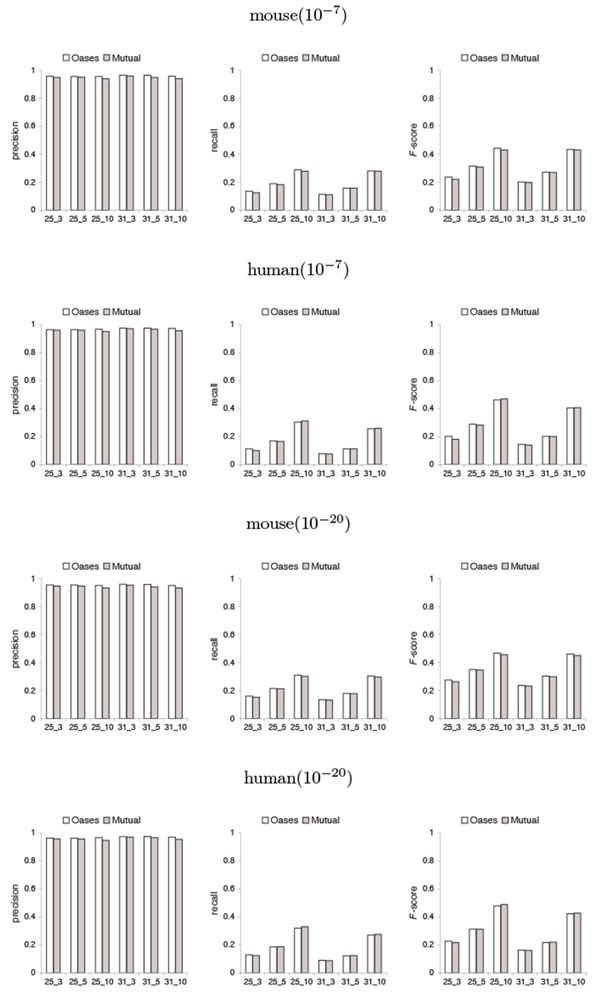
**Precision, recall and *F*-score with respect to the accuracy of shared transcript reconstruction in the test on mouse against human**. Notations are the same as in Figure 5.

By investigating the fitness of the alignment between the predicted shared transcripts and the known transcriptome database sequences, Figures 5 and 6 show that with respect to the accuracy of shared transcript reconstruction, there are tradeoffs between precision and recall by Mutual when compared to Oases. Mutual has slightly lower *F*-scores than Oases in most cases.

### Availability

A software program that implements our algorithm (Mutual) is available at http://faculty.cse.tamu.edu/shsze/mutual.

## Conclusions

We have developed an algorithm that makes use of evolutionary information to simultaneously recover significantly more shared transcripts from RNA-Seq data in two related organisms that may be missed by traditional *de novo *approaches. While more shared transcripts are recovered due to the smaller evolutionary distance between mouse and rat, our algorithm can be applied to related organisms that are evolutionarily farther away, such as between mouse and human.

While known transcriptomes are used as databases during validation, one important characteristic of our algorithm is that no reference transcriptomes or a closely related model organism are needed. Our algorithm can be used to recover shared transcripts that are specific to two closely related non-model organisms, which may not be present in a related model organism that is evolutionarily farther away.

Depending on the size of the de Bruijn graphs, our algorithm can take many processor-hours to run. It takes more than 600 processor-hours to obtain all the predicted transcripts in mouse against rat or in mouse against human for the least stringent values of *k *and the *k*-mer coverage cutoff *c*. Although our algorithm can take much more computational time than the *de novo *algorithms Velvet or Oases, the iterative BLAST searches can be run independently in parallel on a computing cluster. While an additional 60 processor-hours are needed to obtain predicted shared transcripts from the predicted transcripts, a similar procedure is also needed for Oases. No special memory requirement is needed after the de Bruijn graphs are obtained.

One drawback of our algorithm is that only a weak recovery of non-coding regions of mRNA is possible since these regions may not be conserved. Due to the use of similarity information between two related organisms to extend transcripts, our algorithm cannot identify extended transcripts that are not shared between the two organisms.

## Competing interests

The authors declare that they have no competing interests.

## Authors' contributions

AMT and S-HS designed the computational work. SF developed the algorithm and software, did the computational experiments and analyzed the data. All authors wrote, read and approved the final manuscript.

## Declarations

Publication costs for this work were funded by the Open Access to Knowledge (OAK) Fund at the Texas A&M University Libraries.

This article has been published as part of *BMC Genomics *Volume 16 Supplement 11, 2015: Selected articles from the Fourth IEEE International Conference on Computational Advances in Bio and medical Sciences (ICCABS 2014): Genomics. The full contents of the supplement are available online at http://www.biomedcentral.com/bmcgenomics/supplements/16/S11.
